# Triterpenoid Saponins from Washnut (*Sapindus mukorossi* Gaertn.)—A Source of Natural Surfactants and Other Active Components

**DOI:** 10.3390/plants11182355

**Published:** 2022-09-09

**Authors:** Mateusz Sochacki, Otmar Vogt

**Affiliations:** Faculty of Chemical Engineering and Technology, Cracow University of Technology, Warszawska 24, 31-155 Cracow, Poland

**Keywords:** *Sapindus mukorossi*, washnut, triterpenoid saponins, natural surfactants, bioactive compounds

## Abstract

*Sapindus mukorossi* Gaertn., also called the washnut, is a tropical tree of the *Sapindaceae* family. The plant owes its name to its cleaning and washing properties used by the local population as a natural detergent. The most important ingredients of the plant are triterpenoid saponins contained in many parts of the plant, inducing fruits, galls, or roots. The tree also contains other valuable, biologically active compounds that are obtained by extraction methods. Raw or purified extract and isolated saponins are valuable plant products that can be used in the food, pharmaceutical, cosmetic, and chemical industries. This review includes the most important biological and surfactant properties of extracts and isolated saponins obtained from various parts of the plant.

## 1. Introduction

Secondary plant metabolites are a rich source of many substances that manifest biological activity [1]. Contemporary economic development places particular emphasis on pro-ecological activities, including the preference for technological solutions based on natural, renewable material sources, especially using plant sources for this purpose [2,3,4]. Detergents are a key group of products of industrial importance, and they are intended for general use, with a strong impact on the environment [5]. Their surfactant application not only has to do with cleaning agents. Due to the amphiphilic nature of these compounds, which are responsible for adsorption, emulsion, washing, or foaming properties, they are also widely used in the industry [6,7]. Among other things, detergents are products or additives in the food, cosmetic, pharmaceutical, textile-leather, and metallurgical-petrochemical industries [8].

**Figure 1:**
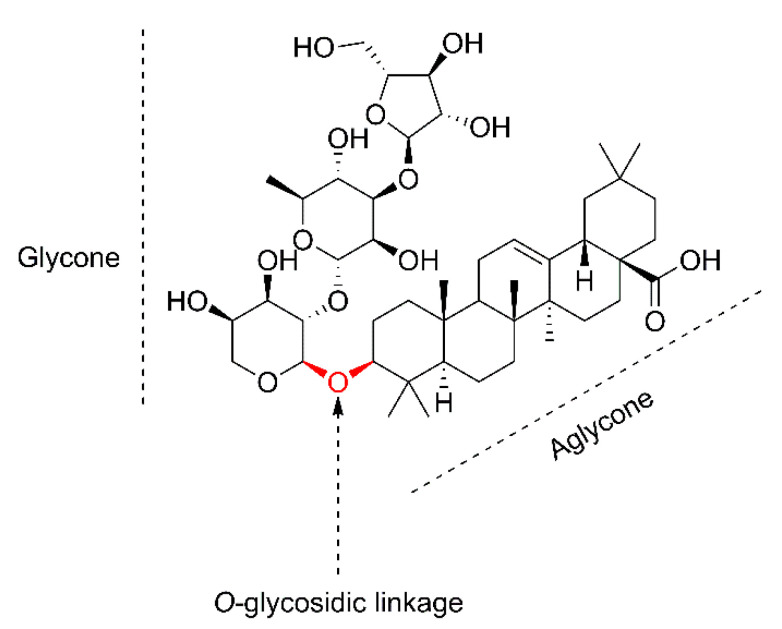
Chemical structure description of oleanolic acid saponin present in the pulp of *S. mukorossi*, isolated by Hu et al. [22].

Saponins are natural, secondary plant metabolites with surfactant properties [9], synthesized by plants and some marine organisms [10]. In terms of chemical structure, they are classified as glycosides. The name saponins is derived from their soap-like properties [11], where the Latin word *sapo* means ‘soap’ [12]. In aqueous solutions, saponins reduce the surface tension of water and manifest foam-forming properties [13]. The detergent properties of saponins result from their amphiphilic structure [14], which consists of a hydrophobic skeleton known as aglycone (or genin) and hydrophilic sugar groups (glycone) [15,16]. The two glycoside-forming parts are the basis for the structural diversion of saponins in nature [11]. The glycone part consists of one or more sugar chains [17], which are then bonded with the aglycone via a glycosidic linkage [18]. The *O*-glycosidic bond separates the two structural parts of saponins [15], functioning as a border (Figure 1). Saponins are mainly classified on the basis of differences in aglycone structure or the number of sugar chains [19]. The basic classification based on the structure of the skeleton distinguishes two main groups: steroid and triterpenoid. Steroid glycoalkaloids are also sometimes included as saponins [10,11]. Steroidal aglycones typically consist of 27, while triterpenoid ones typically consist of 30 carbon units in the skeleton [20]. In addition to carbon variation, the structural diversity of the aglycone involves the different types and arrangements of substituents and further modifications in the backbone [21].

Saponins are found among many families of vascular plants in the form of secondary metabolites [17]. This group also includes representatives of the Sapindaceae family, which synthesize triterpenoid-type saponins [23]. It consists of a number of species, including the *Sapindus* genus: *Sapindus trifoliatus*, *Sapindus saponaria*, *Sapindus laurifolia*, *Sapindus oahuensis,* and *Sapindus mukorossi* [24], discussed further in the present review.

## 2. Plant Description

*Sapindus mukorossi* Gaertn., also called the Chinese soapberry, soapnut, reetha, or washnut, is part of the *Sapindaceae* family [24]. The plant is a deciduous tree found in the tropical and sub-tropical regions of Asia [25], native to China, and cultivated in Japan, India, Bengal, and Pakistan [26]. The tree is a widely cultivated species due to its many applications [27].

### 2.1. Plant Morphology

The tree occupies the upper reaches of the Indo-Gangetic plains, Shivaliks, and sub-Himalayan areas at altitudes of 200–1500 m. In most cases, one can find a tree growing naturally in North India. The plant can reach from 12 to 15 m in height, occasionally reaching up to 20 m and 1.8 m in girth. The trunk is covered with bark of dark-pale yellow, fairly smooth, with numerous vertical line lenticels and fissures exfoliating in irregular wood scales. The tree is covered with leaves (30–50 cm in length), alternate and paripinnate, consisting of 5–10 pairs of leaflets of lanceolate shape, alternate and opposite. Each of the leaflets has a length of 2.5–5 cm. Leaves develop from March or April. At the end of December, they turn yellow and are shed for the period from December to January. For about 2 months (until March), the tree is leafless, then overgrows again. Inflorescences consist of terminal panicles about 30 cm long, with pubescent branches. Numerous greenish-white polygamous flowers, mostly bisexual with five sepals, reach 5 mm across. Flower panicles appear in April with white or purple color. The tree bears fruit in May and matures in June–July. In October and November, ripe fruits change color from yellow-orange to dark brown. The fruits have a spherical shape with one, rarely with two drupels, 1.8–2.5 cm across. Spherical, black seeds reach diameters of 0.8–1.3 cm and are present loosely in the dry fruit [24].

### 2.2. Traditional Plant Applications

Plants of the *Sapindus* genus were utilized by the indigenous people and are now perceived as valuable plant raw materials. Many plant parts of *Sapindus* species are regarded as therapeutic resources, including fruits, bark, roots, seeds, and leaves. These plants are also a source of natural detergents, which have been used to wash silk and wool. Indian jewelers used fruits as a cleaner for precious metal ornaments and to wash out the cardamom. *Sapindus* trees can also be used for phytoremediation, land reclamation, and afforestation [24]. As mentioned, fruits are a valuable resource for the washnut tree [28]. Traditionally in Japan, S. mukorossi pericarps are called enmei-hi, which means ‘life-prolonging pericarp’, and in China, wu-huan-zi, as ‘non-illness fruit’ [29]. In natural medicine, they are used to treat eczema, pimples, psoriasis, epilepsy, chlorosis, migraine, and due to the presence of saponin, also to remove lice from the scalp [27]. Moreover, ground seeds of the soapnut are used to treat problems with dentition, arthritis, colds, nausea, and constipation [26]. In Ayurvedic medicine, seeds were used to remove tan and skin wrinkles [30]. The leaves are used in baths to relieve joint pain and the roots for the treatment of gout and rheumatism [31]. Plants of the genus *Sapindus* are often used for similar purposes. The availability of the species *S. trifoliatus*, *S. Saponaria,* and *S. mukorossi* has contributed to their wide medical use [24].

## 3. Plant Phytoconstituents

The interest in the *Sapindus* species is due to the presence of different saponins in many parts of the plant. *Sapindus* plants also contain many different types of active substances [24,30]. It is assumed that this is due, as in the case of secondary metabolites, to the function they perform in the plant, including mainly ensuring its survival [32].

**Figure 2:**
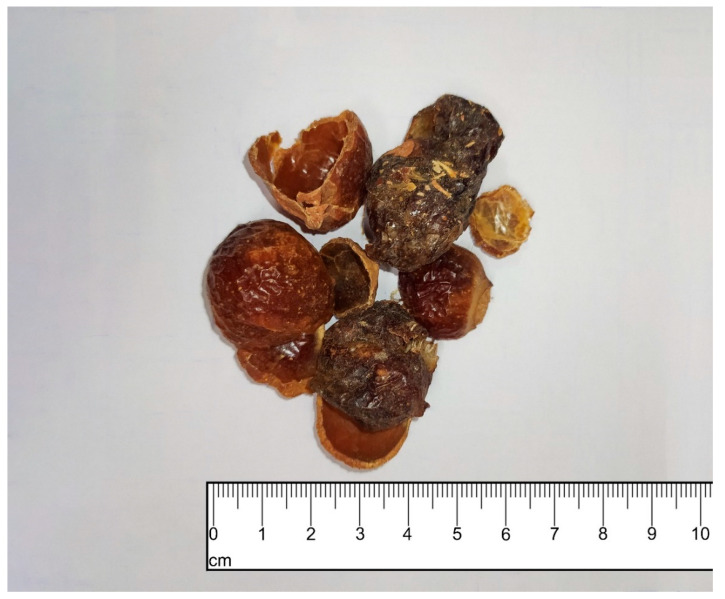
Commercially available, dry washnut pericarps.

**Figure 3:**
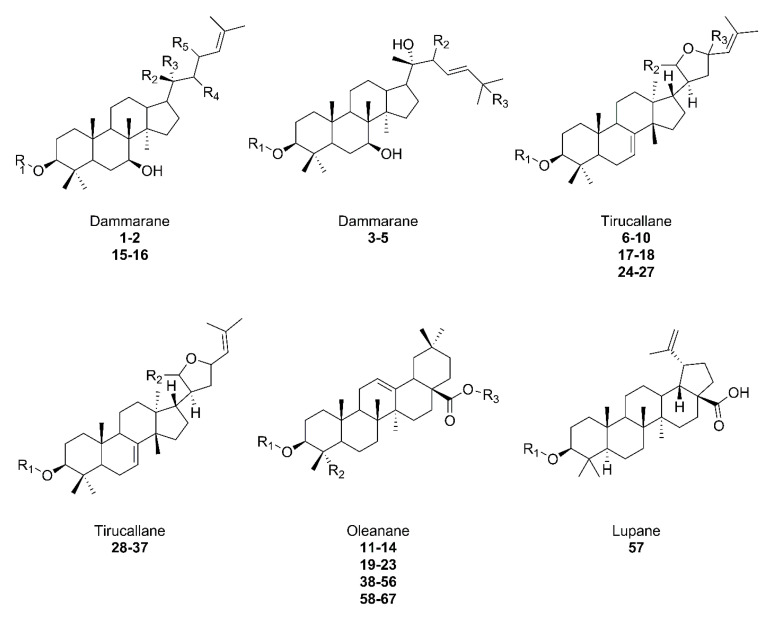
Overview of saponin structures present in *S. mukorossi*. The structure extensions are shown in Table 1 with the corresponding numbers.

Considering the elements of the plant, various phytoactive compounds can be distinguished in the washnut tree. The methanolic extract of *S. mukorossi* leaves contains many bioactive compounds, including alkaloids, flavonoids, phenols, carbohydrates, terpenoids, and saponins [33]. The stems also include flavonoid, phenolic, and polysaccharide constituents [31,34]. A large amount of saponins, amounting to about 10.1–11.5% of the fruit, are present in the pericarp (Figure 2), where this value increases to 56.5% in the drupe [27]. The fruit also contains about 10% sugars, mucilage [35], and sesquiterpene oligoglycosides [36]. Kernel mass consists of 40% oil, which is a mixture of medium-chain monounsaturated and polyunsaturated fatty acids, mostly of oleic and linoleic acid, respectively [28], along with triglycerides [37]. Roots, flowers, and galls are also a source of triterpenoid saponins [38,39,40,41,42,43,44]. The plant is grown for its fruit, the pericarp of which is used as a natural soap. Other parts of the plant are also used for many other purposes [26]. Among the triterpenoid saponins that occur in the plant, three types are most common. Oleanane, dammarane, and tirucallane-type saponins occur in roots, flowers, fruits, pericarps, and galls [38,39,40,41,42,43,44,45,46,47,48], and the recently discovered lupane-type present in the pulp of the plant [22]. The aforementioned structural diversion of aglycone (Figure 3) and glycone part is present in *S. mukorossi*.

## 4. Source of Triterpenoid Saponins

Over the past decades, individual parts of *S. mukorossi* have become the object of research. Various methods are used for the extraction of saponins, followed by isolation by solvent extraction or fractionation and further purification by chromatographic methods [42,49,50,51]. Alternative, ‘greener’ solutions for extracting saponins, such as through fermentation or microwave-assisted extraction, are also being considered [52,53]. 

Studies on the relationship between the saponin skeleton and plant origin have not shown a specific connection between the type of skeleton and the part of the plant containing saponins. One may consider that the concentration of synthesized saponins is related to the development of the plant, its species, and a variety of environmental conditions [15]. Saponin can also affect the proper growth and development of the plant [10]. The structures of the saponin listed below are summarized in Figure 3 and in Table 1 by assignment with respective numbers (**No.**).

Soapnut galls are a rich source of dammarane and tirucallane-type saponins. Kuo et al. [41] analyzed saponins contained in the galls of the plant, confirming the presence of dammarane-type sapinmusaponins A-E (**1–5**). The authors also evaluated the cytotoxic properties of the isolated saponins against human tumor cell lines (Hepa59T/VGH, NCI, HeLa, and Med). Tirucallane-type sapinmusaponins F-J (**6–10**), isolated from the galls, was presented by the team of Huang et al. [40], evaluating their anti-aggregation and cytotoxic activity against platelets. Further studies [38,39] (**11–18**) have shown the presence of sapinmusaponins K-R of oleanane, dammarane, and tirucallane-type in the fruit and galls of the plant. The authors also isolated seven already known analogs (**19–23,41–42**) and further evaluated the antiplatelet and cytotoxic activities of the isolated saponins.

Recent research by the team Wang et al. [42] also confirmed the presence of tirucallane-type sapinmusaponins S-V (**24–27**) contained in the flowers of *S. mukorossi*. Isolated saponins were evaluated for their neuritogenic activity, and the mechanism of action was further elucidated. Tirucallane-type saponins are also present in washnut roots. Rong-Wei et al. [44] isolated tirucallane-type sapimukoside A and B (**28,29**) from the roots of the plant. Further research of the roots performed by Ni et al. [43,45] demonstrated the presence of tirucallane-type sapimukosides C-J (**30–37**).

Various parts of the *S. mukorossi* fruits are a source of oleanane-type saponins. The team of Nakayama et al. [47] isolated oleanane-type bisdesmosidic saponins (**38–40**) from plant pericarps. The authors also analyzed the solubilization properties of the isolated saponins. Mukurozi-saponins Y_1_ (**38**), Y_2_ (**39**), and X (**40**) greatly increased the water solubilities of monodesmosidic analogs. Chirva et al. [54,55,56,57] isolated a number of oleanane-type saponins from soapnut fruit by silica gel chromatography in different eluent ratios. The discovered sapindosides A-D (**41–44**) contained hederagenin as backbones and one sugar chain, while sapindoside E (**45**) hederagenin contained two sugar chains. Zhang et al. [58] isolated a new oleanane-type saponin (**46**) and a new oligosaccharide, along with known analogs (**11,47**), from the n-buntanol extract of the fruit. The new saponin structure was evaluated as hederagenin-3-*O*-β-d-glucopyranosyl-(1→3)-β-d-xylopyranosyl-(1→3)-β-d-xylopyranosyl-(1→3)-α-l-rhamnopyranosyl-(1→2)-α-l-arabinopyranoside. In a later study of the plant fruit, the team of Zhang et al. [46] isolated two new glycosides, sapindoside G (**48**) and 4″,4′′′′′-*O*-diacetylmukurozioside IIa, and two saponins, hishoushi-saponin Ee (**21**) and sapindoside A (**41**). The authors evaluated their inhibitory activity against human lung adenocarcinoma cells A549.

Huang and associates [59], as part of their evaluation of the molluscicidal activity of saponins from the plant pericarp, isolated a new acylated saponin called hederagenin 3-*O*-(2,4-*O*-di-acetyl-α-l-arabinopyranoside)-(1→3)-α-l-rhamnopyranosyl-(1→2)-α-l-ara-binopyranoside (**49**) and six previously known analogs (**19**,**21**,**22**,**42**,**47** and **50**). Other new acylated derivatives were isolated from the pericarp by Sharma et al. [48,50] named hederagenin-3-*O*-β---xylopyranosyl-(2→1)-[3-*O*-acetyl-α-l-arabinopyranosyl]-28-*O*-α-l-rhamnopyranosylester (**51**) and hederagenin 3-*O*-α-l-rhamnopyranosyl (3→1)-[2,4-*O*-diacetyl-α-l-arabinopyranosyl]-28-*O*-β-d-glucopyranosyl-(2→1) [3-*O*-acetyl-β-d-glucopyranosyl] ester (**52**). 

New saponins were isolated from the pulp of *S. mukorossi* by the team Hu et al. [22], then evaluated for antifungal activity. The authors isolated four previously undescribed oleanane-type saponins and one lupane-type, oleanolic acid 3-*O*-α-l-arabinofuranosyl-(1→3)-α-l-rhamnopyranosyl-(1→2)-α-l-arabinopyranoside (**53**), hederagenin 3-*O*-5‴-*O*-acetyl-α-l-arabinofuranosyl-(1→3)-α-l-rhamnopyranosyl-(1→2)-α-l-arabinopyranoside (**54**), 23-*O*-acetyl-hederagenin 3-*O*-β-d-xylopyranosyl-(1→3)-α-l-rhamnopyranosyl-(1→2)-α-l-arabinopyranoside (**55**), gypsogenin 3-*O*-α-l-arabinopyranosyl-(1→3)-α-l-rhamnopyranosyl-(1→2)-α-l-arabinopyranoside (**56),** and betulinic acid 3-*O*-β-d-xylopyranosyl-(1→3)-α-l-rhamnopyranosyl-(1→2)-α-l-arabinopyranoside (**57**), respectively. Ling et al. team [36] also evaluated the chemical composition of the major components present in *S. mukorossi* fruit using HPLC-ESI-QTOF-MS/MS. By applying this method, the authors discovered potential new chemical compounds, including 9 acyclic sesquiterpene oligoglycosides and 8 triterpenoid saponins (**58–65**).

Significant developments in analytical techniques and methods of analyzing organic compounds have resulted in the discovery of new triterpenoid saponins. Several of the previously mentioned authors’ work on the structural identification of saponins, was also developed into an evaluation of the biological activity of the isolated compounds. 

## 5. Biological Activity of Saponins, Extracts, and Plant Oil

The biological activity of saponins is linked to their role in plant organisms. Although the role of saponins is not fully understood, it can be indicated that their function in plants is primarily protective [13]. Mugford et al. [11] emphasize that the observed antimicrobial, anti-herbivore and insecticidal properties may perform the plant’s protective functions. Moses et al. [10] also developed this theory to include regulatory functions for the plant itself. Saponins can be synthesized and accumulated in various plant parts, acting as phytoanticipins. The content of saponins may vary in response to abiotic as well as biotic stresses, confirming their protective role [60]. The amount of saponins in plants is also related to the stage of development, growth, and maturation of the plant [15]. 

Saponins are biologically active plant metabolites widely distributed in nature [17,61]. Many sources show a number of interesting biological properties of saponins. Many of them are related to the potential protective mechanism of plants, some in reference to animal organisms [62]. Hemolytic activity of saponins toward erythrocytes has been noted, through the interaction with sterols present in the membrane [23,63]. In addition, antibacterial [64,65], antifungal [66,67], or antiviral [12,62] properties have also been documented against saponins obtained from various plant species. The most common pharmacological properties of saponins include anti-inflammatory [51,68], anticancer, cytotoxic [10,20,69], antioxidant [70,71], molluscicidal [59,72], insecticidal [73,74], and antiparasitic [49,75] properties. The most relevant biological properties will be further described in terms of practical industrial applications.

### 5.1. Antibacterial Activity of Plant Extracts

A bactericidal assay of the *Sapindus saponin* water extract was conducted by Heng et al. [76]. The assay showed inhibitory activity against bacterial strains such as *Salmonella paratyphi A* (CMCC 50095), *Shigella dysenteriae* (CMCC 51334), *Listeria welshimeri* (ATCC 35897), *Escherichia coli* (ATCC 8099), *Pseudomonas aeruginosa* (ATCC 15442) and *Staphylococcus aureus* (ATCC 6538) for MIC values of 25 mg/mL and the corresponding diameters of inhibition zones. Another evaluation of the bactericidal properties of washnut pericarp extracts represents the work of Sağlık et al. [77], in which the effect of using different solvents to obtain this extract was determined in microbiological tests conducted by the DDA, BMA, and ADA methods. The lowest MIC against *Porphyromonas gingivalis* (ATCC 33277) and *Actinomyces odontolyticus* (clinical isolate) was demonstrated by the methanol and butanol extract (0.01 mg/mL), and the methanol and ethanol extract (1.28 mg/mL), respectively. All extracts showed inhibitory activity against *Fusobacterium nucleatum* (ATCC 25586) for MIC values of 10.24 mg/mL. A study conducted by the team of Ibrahim et al. [35] reported inhibitory effects on the growth of *Helicobacter pylori* via a disk diffusion method in vitro and in vivo tests performed on male Wister rats. A total of 30 resistant clinical isolates of *H. pylori* and four microorganisms (*Staphylococcus aureus*, *Bacillus subtilis*, *Escherichia coli,* and *Proteus vulgaris*) were also tested for their susceptibility patterns against plant extracts. The screening demonstrated the sensitivity of all 30 isolates and four organisms against the test compound. Ethanol and chloroform extracts from the pericarp showed inhibition at concentrations of 10 µg/mL in the in vitro study against the isolates. In the in vivo study, *H. pylori* infection was cleared with a 2.5 mg/mL dosage of *S. mukorossi* extract administered orally for seven days. In addition, *H. pylori* did not gain resistance after 10 consecutive passages. Another in vitro study, conducted by Sharma et al. [48] on ethanolic pericarp extracts determined by the agar well diffusion method showed significant inhibitory activity at 50 µg/mL and 100 µg/mL against *Esherichia faecalis*, *Pseudomonas aeruginosa*, *Staphylococcus aureus*, *Alcaligenes denitrificans*, *Klebsiella pneumoniae*, *Bacillus cereus*, *Pseudomonas alcaligenes*, *Micrococcus luteus* and *Bacillus subtilis* strains.

### 5.2. Antifungal Activity

#### 5.2.1. Activity of Plant Extracts

*Sapindus saponin* water extract showed inhibitory activity against *C. albicans* (ATCC 10231) and *T. rubrum* (ATCC 294) strains for MIC values of 0.78 mg/mL and the corresponding diameters of inhibition zones [76]. The aqueous pericarp extract showed inhibitory activity against *Venturia inaequalis* and *Botrytis cinerea* with a relative percentage of 100% and 54% at a concentration of 30,000 ppm, respectively. Chloroform-methanol extract from the pericarp showed identical activity against *V. inaequalis* and increased (74%) activity against *B. cinerea* at 30,000 ppm [78]. Sağlık et al. [77] presented the results of antifungal inhibition of pericarp extracts obtained with various solvents against *C. albicans* from various sources (ATCC 10231, clinical isolates 1-3). High inhibition activity was shown against ATCC 10231 strain by a butanol extract at 0.2 mg/mL MIC. For other isolates, MIC values ranged from 0.2 to 0.4 mg/mL against all extracts.

#### 5.2.2. Activity of Isolated Saponins

An antifungal assay was conducted by the team Hu et al. [22], which presented the inhibition of isolated oleanane-type saponins from the pulp of *S. mukorossi* on *Trichophyton rubrum* (ATCC 28188) and *Candida albicans* (SC5314) strains. In vitro tests of oleanolic acid 3-*O*-β-d-xylopyranosyl-(1→3)-α-l-rhamnopyranosyl-(1→2)-α-l-arabinopyranoside (**66**) showed inhibitive activity MIC_80_ at 8 μg/mL against *T. rubrum*, while oleanolic acid 3-*O*-α-l-arabinopyranosyl-(1→3)-α-l-rhamnopyranosyl-(1→2)-α-l-arabinopyranoside (**67**) showed MIC_80_ at 8 μg/mL against to *T. rubrum* and *C. albicans.*

### 5.3. Anti-Inflammatory Activity

#### 5.3.1. Activity of Plant Extracts

Shah and associates [31] evaluated the activity of methanol extract of S. mukorossi stem bark and its fractions. In vitro studies demonstrated the inhibitory effects of the extract and fractions on heat-induced protein denaturation. The aqueous fraction SMA showed the best activity of 82% inhibition for a 500 μg/mL concentration. The methanolic extract of SMM caused 43% inhibition at a concentration of 500 μg/mL. As a reference substance, the authors used loprin, a standard drug, which showed inhibition of 79% at 500 μg/mL. The extracts and fractions were tested in vivo with carrageenin-induced paw edema assay in Sprague-Dawley rats versus the standard, which was diclofenac potassium. SMB and SMA fractions showed a strong percentage of edema inhibition of 73.43% and 84.19%, respectively, after 3 h at a dose of 300 mg/kg. Diclofenac showed 73.99% inhibition after 3 h at a dose of 10 mg/kg. The team of Deng et al. [52] also carried out an evaluation of the anti-inflammatory properties of washnut saponins against gout disease, in the form of an analysis of xanthine oxidase inhibitory activity. In addition, the authors developed an efficient, environmentally friendly and reliable microwave-assisted extraction (MAE) technique of saponins from the *S. mukorossi* pericarp. Xanthine oxidase is a key enzyme of uricogenesis, which results in inflammation as gout. The in vitro studies of washnut saponins presented a strong inhibitory activity (89.87%) at a concentration of 100 μg/mL against xanthine oxidase. The result was very close to the inhibition of the control drug, allopurinol (92.25%), tested at the same concentration. On the basis of the above-mentioned studies, the anti-inflammatory activity of S. mukorossi can be proved.

#### 5.3.2. Activity of Isolated Saponins

Studies conducted on female Wistar rats by the team of Takagi et al. [79] concerned carrageenin-induced hind paw edema, granuloma pouch and adjuvant arthritis. The authors examined the effects of hederagenin and crude saponin isolated from *S. mukorossi* on the above-mentioned conditions. Results showed positive effects of crude saponins on carrageenin edema when administered orally and by intraperitoneal injection, while hederagenin and the other substances showed anti-inflammatory properties only when injected. Hederagenin administered orally at 100 and 200 mg/kg per day for 7 days revealed no significant inhibitory effect on granuloma and exudate formation, while the crude saponin at the same dosage and administration showed a significant effect.

### 5.4. Antioxidant Activity of Plant Extracts

Natural antioxidant properties are highly valued due to the damaging role of free radicals on health and the quality of everyday products [80]. Extracts from *S. mukorossi* exhibit antioxidant properties. Shah et al. [31] performed antioxidant assays of the stem bark extract and fractions, such as scavenging of DPPH, hydroxyl radical, nitric oxide and other evaluations of antioxidant potential. Results with the highest DPPH sweeping activity IC_50_ valued for 162.5 μg/mL of the methanol extract (SMM) in relation to the IC_50_ of ascorbic acid valued for 89.2 μg/mL. The hydroxyl radical scavenging activity IC_50_ was 44.7 μg/mL of the ethyl acetate fraction (SME) in relation to the rutin standard IC_50_ of 21.35 μg/mL. For the nitric oxides scavenging activity, the IC_50_ was 152.9 μg/mL for the ethyl acetate fraction (SME) versus the IC_50_ of 65.92 μg/mL for the ascorbic acid standard. The authors also quantified the total phenolic and flavonoid content in all the extracts/fractions. In the studies of the Chen I C et al. team [81], the antioxidant effect of saponin extract obtained from washnut fruits was presented. The best percentage result of the DPPH radical sweeping activity was 91.56%, obtained by the process optimization using Response Surface Methodology (RSM). Chen C-Y et al. [80], also evaluated the DPPH radical scavenging capacity of extracts obtained with methanol, ethyl acetate and hexane at specific concentrations. The percentage sweeping activity resulted with 10 μg/mL concentrations were 34%, 13% and 4.6% for methanol extract, ethyl acetate and hexane, respectively. The results were related to ascorbic acid (100 μM) as a control compound of 100% activity. The authors also assessed the antimicrobial, antiproliferative activity against various human cancer cells and tyrosinase inhibition activity of *S. mukorossi* extracts.

### 5.5. Molluscicidal Activity of Plant Extracts

The washnut pericarp extract showed strong molluscicidal activity against Golden Apple snails, *Pomacea canaliculata*. The molluscicidal assay performed by means of the submersion method showed activity against *P. canaliculata*. The LC_50_ values were 85, 22 and 17 ppm after 24, 48 and 72 h exposure times, respectively. A field assay showed that spraying a rice field with a soapnut extract powder in a concentration of 4 ppm resulted in mortality rate of 62% after the 72 h treatment. The results were compared to control pesticides [59]. The Upadhyay et al. team [82], also reported molluscicidal activity of plant extracts against *Lymnaea acuminata* snails. The ethanolic extract from the pericarp of *S. mukorossi* showed the highest activity in relation to other solvents after 24 h with a biocidal value LC_50_ of 2.75 mg/L. The column-purified fraction of *S. mukorossi* showed activity after 96 h equal to LC_50_ 5.43 mg/L.

### 5.6. Antipyretic Activity of Plant Extracts

The results of Shah et al. [31] research on the extract and fractions obtained from the steam bark of *S. mukorossi* showed antipyretic properties on Sprague-Dawley rats with brewer’s yeast-induced pyrexia (*Saccharomyces cerevisiae*). Strong antipyretic properties were exhibited by the aqueous (SMA) and butanol (SMB) fractions for a concentration of 300 mg/kg, reducing the rectal temperature of rats after 4 h of administration, from 37.11 °C to 35.21 °C and 37.5 °C to 35.54 °C, respectively. The remaining extracts/fractions also lowered the rectal temperature depending on the concentration and time.

### 5.7. Analgesic Activity of Plant Extracts 

Shah and associates [31] also evaluated extracts/fractions from the stem bark of *S. mukorossi* in terms of analgesic properties based on the hot plate (latency time) test against male rats of Sprague-Dawley, in reference to a standard drug, loprin. All samples showed an increase in latency times at 0, 30, 60 and 120 min of analgesia induction, which depended on the concentration of the active substance. The aqueous fraction (SMA) showed strong analgesic activity at 300 mg/kg with the precent analgesia of 2.21, 23.97, 45.64 and 55.78 at 0, 30, 60 and 120 min, respectively. The authors related the results loprin at 10 mg/kg and the percent analgesia of 4.5, 36.35, 59.64 and 65.64 at 0, 30, 60 and 120 min, respectively.

### 5.8. Insecticidal Activity

#### 5.8.1. Activity of Plant Extracts

Insecticidal activity was evaluated by the team Eddaya et al. [83]. The authors assessed the effect of the aqueous extract of washnut pericarp on the development of a spearmint pest, a moth *Thysanoplusia orichalcea*. The extract was applied to spearmint leaves, which were the food of *T. orichalcea* larvae in increasing concentrations of 0.625%, 1.25%, 2.5%, and 5.0% (*w/v*) for 7 days. The addition of pericarp extract reduced larval weights by 7% to 68% and delayed larval development by 1 to 2 days. Furthermore, the larval consumption was reduced between 40% and 100% depending on concentration, exposure time and stage used. 

#### 5.8.2. Activity of Isolated Saponins

Saha et al. [74] reported the feeding deterrent and insect growth regulatory activity of triterpenic saponins and sapogenins against *Spodoptera litura*. Saponins were extracted and separated from the pericarp of soapnut and then hydrolyzed to obtain sapogenins. Saponins and sapogenins were applied on castor leaves constituting food for *S. litura*. The highest antifeedant activity of 48.2% was achieved using solutions with a saponin concentration of 1%. At the same concentration, a higher activity value of 51.2% was achieved for acid hydrolyzed saponins. The second parameter studied by the authors was the regulatory effect on *S. litura* growth of 42.2% for saponins and 69.5% again after acid hydrolysis for 1% concentrations.

### 5.9. Antitumor Activity

#### 5.9.1. Activity of Plant Extracts

Studies on the aqueous extract (SaM) performed by Liu et al. [34] also demonstrated antitumor activity against A549 cells despite the lack of detectable saponins in the leaf and stem extract. The polysaccharide-rich SaM extract reduced the proliferative potential of lung adenocarcinoma cells, induced intracellular oxidative stress and necrotic cell death. In addition, exposure to SaM attenuated cell migration. Based on the in vivo results on model animals bearing LL/2 tumor cells, the authors demonstrated antitumor properties of the extract, which did not cause any unwanted organ damage, immunotoxicity and off-target inflammation. 

#### 5.9.2. Activity of Isolated Saponins

Some of the saponins of the washnut have shown anticancer properties. Research in this regard was conducted by Kuo et al. [41], who evaluated the cytotoxic activity of dammarane-type triterpenoid saponins isolated from *S. mukorossi* galls. The tests were conducted against human cancer lines (Hepa59T/VGH, NCI, HeLa and Med). The cytotoxicity of sapinmusaponins A, C, D and E (**1,3-5**) were shown to be moderate within ED_50_∼9-18 µg/mL. Another evaluation of anticancer properties by Zhang et al. [46] was performed with the newly isolated glycosides and saponins from the fruit of *S. mukorossi* conducted against human A549 lung adenocarcinoma cells. Sapindoside G (**48**), 4′′,4′′′′′-*O*-diacetylmukurozioside IIa, hishoushi-saponin Ee (**21**) and sapindoside A (**41**) showed inhibitory effects, within 69.2% and 83.3% against A549 cells at a concentration of 100 μg/mL. The compounds were able to arrest cell growth by causing cell apoptosis, due to caspase-3 activation.

### 5.10. Cutaneous Activity

#### 5.10.1. Activity of Plant Extracts

Washnut seeds were used in Ayurvedic medicine to remove tans and freckles from the skin [30]. Wei et al. [61] evaluated the protective effects of *S. mukorossi* extract in the function of freckle removal and skin brightening, also predicting an potential anti-acne mechanism. The authors obtained the saponin fractions through a multi-step purification process of the aqueous soapnut fruit extract, which included fermentation, ethyl acetate extraction and semi-preparative HPLC purification. One of the goals of the study was to evaluate the antimicrobial effect of the fraction against the bacterium *Propionibacterium acnes*, which is the main cause of inflamed lesions in acne vulgaris. Among the seven fractions obtained in the work [61] trough semi-preparative HPLC, the F4 fraction showed the highest activity against *P. acnes* at MIC of 0.06 mg/mL. In addition, the SWF and F4 fractions showed highest inhibition activity against tyrosinase at 2 mg/mL were 63.88% and 67.26%, respectively. Tyrosinase is a key enzyme responsible for melanin biosynthesis among plant and animal organisms. Both of the above-mentioned fractions also presented inhibition activities on *P. acnes* lipase, but the F4 fraction displayed stronger anti-lipase activity by 1.60-fold in comparison to SWF fraction at a concentration of 2.0 mg/mL. Both fractions have potential as acne treatments or skin whitening agents, in favor of the F4 fraction. Another evaluation was conducted by Chen C-Y et al. [80], who assessed the inhibitory effect on tyrosinase with *S. mukorossi* seeds extracted with three solvents, methanol, ethyl acetate and hexane. An in vitro assay was performed against mushroom tyrosinase at extract concentrations of 10 μg/mL. The results were related to kojic acid (100 μM) as a positive control of 100% activity. Methanol and the ethyl acetate extract showed minor tyrosinase inhibition, 17.8% and 12.3%, respectively. The hexane extract did not show any inhibitory activity. The authors suggested that the reason for the activity of the methanol extract is due to polar polyphenols and flavonoid constituents, soluble in methanol.

#### 5.10.2. Activity of Plant Oil

The composition and effect of seed oil on wound healing rate was conducted by Chen C-C and associates [37]. *S. mukorossi* oil was extracted from plant seeds and evaluated via an in vitro and an in vivo test. Based on the results of the in vitro scratch assay, seed oil increased the migration of CCD-966SK cells toward the scratched area. The wound closure rate of oil-treated cells was 24.73% higher than control cells after 6 h, which was 6.45%. CCD-966SK cells treated with oil showed significantly increased healing ability compared to control cells. In addition, after 24 h of culture, the oil-treated cells had a wound closure width of 82.79% compared to 44.08% of the control group, which was 1.88-fold greater. The authors conducted in vivo studies in reference to healthy male Sprague-Dawley rats, whose wounds were covered with oil-hydrogel. Healing effects were observed for 12 days. The wounds in the oil-treated group healed faster with the growth of granulation tissue, absence of edema, and lower secretions in comparison with the untreated control group. Quantitative evaluation presented that in vivo experimental wounds treated with seed oil showed a statistically greater reduction in wound area (74.14%) compared to that of the untreated control wound (91.02%). Despite this, the authors stress that the skin wound healing activity of *S. mukorossi* seed oil has not been fully confirmed.

### 5.11. Saponin Toxicity

In connection with the evaluation of saponins in reference to their biological properties, the studies also focus on assessing their toxicity. The team Wei et al. [61] evaluated the toxicity of saponins present in the F4 fraction extracted from the washnut fruit by a multi-step purification process. A toxicity assay was performed on three organisms using the T.E.S.T. software. The authors evaluated acute toxicity (oral LD_50_ on rats, LC_50_ on fathead minnow after 96 h, and LC_50_ on *Daphnia magna* after 48 h), mutagenicity, developmental toxicity, and bioaccumulation factors of compounds present in the F4 fraction. Based on the results, four saponins contained in the F4 fraction (Rarasaponin II (**11**), Rarasaponin VI (**14**), Mukurozisaponin E_1_ (**22**), and Mukurozisaponin G (**19**)) were classified as at least harmful (<100 mg/L) to fathead minnow and *D. magna*. The compounds showed no harm to rats for oral LD_50_ between 115.58 and 238.76 mg/kg, indicating that the same substances exhibit different toxicity to different organisms. The reason for the high toxicity of aquatic organisms tested may be due to the piscicidal activity of *S. mukorossi* [27].

Another evaluation of the toxicity of saponins was undertaken by the team of Du and associates [84]. The saponins were extracted from the pulp of *S. mukorossi* in 85% ethanol, followed by washing with chloroform and ethyl acetate. The dry matter was re-dissolved in 85% ethanol and centrifuged. The separated supernatant was used as the saponin extract. Toxicity evaluation was carried out against SPF Wistar rats. Acute oral toxicity study showed that LD_50_ of soapnut saponins after administration was equal to 9260 mg/kg and 7940 mg/kg for female and male rats, respectively. An acute dermal toxicity study showed that the LD_50_ of saponins is greater than 5000 mg/kg in both male and female Wistar rats. A dermal irritation test indicated an average irritation score per day of each rabbit equal to zero after 14 days of continuous irritation. On the basis of the results and the cosmetic toxicity classification standard, the authors concluded that saponins from *S. mukorossi* are safe for use in cosmetics. A skin irritation study of saponins was also conducted by Wei et al. [61]. Two fractions of F4 and SWF extracted from *S. mukorossi* fruit by the multi-step purification were tested with a 4-hr human patch test. Among the 30 subjects tested, a concentration of 25 mg/mL of the F4 fraction and SWF did not cause swelling or allergic reaction in any of the test subjects. On the basis of the study, the authors suggests that the toxicity of the saponins should not be ignored. Further toxicological evaluation is required.

## 6. Surface Activity

As mentioned earlier, saponins are characterized by surfactant properties that allow them to lower the surface tension of aqueous solutions [12,17,85]. This feature is a direct result of the saponin structure, since they consist of two parts with different solubility in water, which in effect form an amphiphilic molecule [9,20]. Saponins in aqueous solutions assume the form of monomers, adsorbing at the water-air interface [13,68], and after reaching critical micelle concentration (CMC), they begin to aggregate into micelles [8], [15,86]. Saponins exhibit many functional properties, such as foam formation [87,88], as well as wetting [88,89], emulsifying [25,90], solubilizing [91,92], adsorptive [19,93] and detergent properties [14,94].

### 6.1. Surface Tension

Yang C-H et al. [25] evaluated the surface-active properties of saponins extracted from the pericarp of soapnut. The crude extract was obtained by 3-fold extraction of the raw material with boiling water, followed by filtration, concentration, and partitioning with ethyl acetate and n-butanol. The authors, therefore, obtained a saponin extract, which, for a concentration of 0.5%, was compared with synthetic surfactants in an aqueous medium. The surface tensions of 0.5% solutions of the saponin extract, SDS, and Tween 80, respectively, were 51.7 mN/m, 35.6 mN/m, and 41.7 mN/m, compared to water tensions of 72 mN/m. Therefore saponins have potential as a detergent. Similar results of S. mukorossi pericarp extract were obtained by Wojtoń et al. [89]. In their study, the minimum surface tension value of the extract reached ca. 52 mN/m in distilled water. Better results were obtained by the team Ghagi et al. [95], reporting a minimum surface tension value of 38 mN/m for *S. mukorossi* pericarp extract. The surface tension of the extracted surfactants from washnut pericarp performed by Pradhan et al. [14] reached a value of 35.30 mN/m.

### 6.2. Foaming Properties

Another parameter studied by Yang C-H et al. [25] had to do with the foam properties of soapnut crude saponin extract. The authors obtained the highest foam height value for the aqueous 0.5% SDS solution, followed by 0.5% Tween 80 and 0.5% crude saponin extract, for which the heights were similar. The saponin extract foaming height was 65% of the height obtained for SDS. These results showed that the 0.5% crude saponin extract solution had sufficient foaming power. The foam height did not change significantly over 5 min. The R5 value of 0.5% saponin solution was 91.7%, reflecting suitable foam stability. The foam stability of the saponin extract from the pericarp of *S. mukorossi* was also evaluated by Yekeen and associates [88]. CO_2_ gas was used as the foam generator by Teclis Foamscan equipment at room temperature and at 60 °C, using SDS as a reference. At room temperature, a saponin solution of 0.4 wt.% showed the most stable foam. In the case of the reference surfactant, the SDS solution of 0.2 wt.% showed the greatest level of stability. On the basis of the above-mentioned studies, the authors concluded that saponins generate more stable foam at room temperature than SDS. Although the saponins presented a slightly lower foam stability at 60 °C, the optimum concentration of saponins, in this case, was lower than under room conditions and accounted for 0.1 wt.%. The results indicate that saponin-stabilized foam can be generated at high temperatures, remaining fairly stable before collapsing.

### 6.3. Wetting Properties

Yang C-H et al. [25] also presented the wetting properties of a pericarp plant extract. The evaluation was to measure the time required for the liquid to penetrate deep into the gray cotton yarn by replacing the air contained inside. At a concentration of 0.5%, the wetting time was 0.1 min for SDS and 3 min for *S. mukorossi* extract. This implies that the wetting properties of the tested extract are weaker than those of SDS. A different approach for wetting ability evaluation was presented by Wojtoń et al. [89]. The measurements were carried out against the hydrophobic PTFE material by determining the surface contact angle of the aqueous pericarp extract of the plant. According to the results, the wetting angle decreases in response to the increasing surfactant concentration. The lowest angle value of 102.6° was obtained at 8 g/L of the *S. mukorossi* extract concentration. For Triton X-100 and X-165 non-ionic surfactants, the contact angle values on PTFE were 68° and 78°, respectively. Another evaluation of wettability was conducted by the team of Yekeen et al. [88]. The authors determined the contact angles of the saponin extract from the pericarp of *S. mukorossi* against the surface of two types of shale and aged Berea sandstone. The obtained results were compared to the wetting angles obtained from commercial SDS and deionized water on the same surfaces. The contact angle on the shale 2 was reduced from a value of 52.44° to 38.3° for 0.01 wt.% and to 8.85° for 1.0 wt.% concentration of the saponin extract. In comparison, the value of 52.44° of contact angle was reduced to 19.66° for 0.5 wt.% SDS. The results indicate that the saponin extracted from soapnut be considered as fair EOR agent (enhanced oil recovery) for altering the wettability of an unconventional shale reservoir.

### 6.4. Critical Micelle Concentration

The critical micelle concentration of saponins determines the concentration value at which monomers aggregate into bigger structures, micelles [13]. According to the study by Wojtoń et al. [89], the CMC of pericarp extract in deionized water was 0.32 g/L (0.032 wt.%) in 20 °C. Another evaluation by Yekeen et al. [88] showed the CMC value at a pericarp extract concentration of 0.2 wt.% Ghagi et al. [95] determined the CMC at 0.017 g/mL (1.7 wt.%) of the *S. mukorossi* pericarp extract. In a study conducted by Balakrishnan et al. [86], the CMC of a *Sapindus* saponin extract was determined for a concentration of 0.045 wt.%. The authors also evaluated the effects of pH, water hardness and temperature on the CMC of the extract. The CMC value decreased with increasing temperature and salt concentration, while it increased with raising water hardness and pH. Pradhan and associates [14] evaluated the micellization properties of surfactants from the outer pericarp at 0.0075 g/mL. Another measurement of CMC was carried out by Mondal et al. [96] of saponins isolated and purified from the washnut pulp. The authors determined CMC via UV-Vis method equal to 0.054 mmol/L in 35 °C and a surface tension equal to 38 mN/m.

### 6.5. Solubilizing and Emulsifying Properties

Balakrishnan et al. [86] evaluated the solubility of two types of crude oils and vegetable oil in solutions of *Sapindus* saponins versus synthetic surfactants (SDS and Triton X100) using a micellar solubilization technique. At low concentrations of the surfactant, the micellar solubilization of crude oils in saponins was better compared to synthetic surfactants, while the opposite effect occurred with vegetable oil. Ghagi and associates [95] reported the emulsion properties of *S. mukorossi* extracts. The study were conducted in relation to kerosene and various plant oils enriched with aqueous ritha solutions, in comparison with commercial SDS. The emulsification activity of ritha and SDS solutions with kerosene were similar, reaching approximately 67%. The authors emphasize suitable emulsifying properties of ritha, compared to SDS. Another evaluation of the emulsion properties of saponins from the outer pericarp was carried out by preparing an emulsion with 20 mL of refined oil and 20 mL of surfactant solution. Ritha emulsion remained stable for almost 2 h. Stability increased with surfactant concentration until CMC was reached, then decreased dramatically and improved again with increasing concentration. According to the authors, the reason may be the decreasing amount of saponins capable of adsorption at the water-oil interface in response to micelle formation. Further increase in surfactant concentration may lead to emulsion breakdown due to rapid droplet coalescence. The results were related to Henko, a synthetic ionic surfactant, which performed more poorly compared to natural surfactants from *S. mukorossi* [14]. Results prove that saponins extracted from *S. mukorossi* plant can substitute synthetic emulsifiers.

### 6.6. Washing Properties

The washing properties of the substance were tested by Shi and associates [97]. Soapnut fruit peels served as raw material in microwave-assisted radiation during aqueous extraction. The crude extract was subjected to electron-beam and Co^60^-γ irradiation. The authors evaluated the effect of irradiation on total saponin content and saponin washing properties on bleached cotton cloth stained with tomato and lemon juice, snow chrysanthemum extract, carbon ink and soy sauce. Dirty clothes were placed in glass bottles filled with 100 mL extracts diluted to 3% volume fractions for 12 h, stirring every hour. After drying the cloths, washing effect was evaluated according to a point scale of stains visibility. The detergent ability of the non-irradiated crude extract was the lowest. The effect of electron radiation at 6 kGy contributed to the highest improvement in washing ability. The effect of Co^60^-γ gamma radiation caused no apparent change in the detergent capacity. The results are due to the total amount of saponins contained in the extracts. Electron radiation increased this value. Total washing capacity of crude extracts was between 300 and 450 points on a scale, where 500 meant complete stain removal and 0 meant no washing effects. The authors also evaluated the antibacterial activity of the extracts. Pradhan et al. [14] demonstrated the washing ability of saponins extracted from the outer pericarp of *S. mukorossi*. The authors prepared a simulated dirt in which they immersed a cotton Poplin cloth, then dried and weighed the cloth. The cloth was immersed in the saponin solution for 10 min, rinsed with water, dried and weighed to evaluate the washing ability. The results were related to the synthetic surfactant, Henko. Ritha saponins washed out about 20% to 80% of the grime from the cloth, compared to about 30% to 75% washed out by Henko, in terms of increasing surfactant concentration. A different approach to assessing detergent properties was evaluated by Yang C-H et al. [25]. Washed and dried hair samples from a beauty salon were cut, then soaked in a sebum solution as a mixture of oily substances (olive oil 20%, coconut oil 15%, stearic acid 15%, oleic acid 15%, paraffin wax 15% and cholesterol 20%) dissolved in hexane for 15 min with intermittent shaking. After evaporation of the solvent, the swatch was weighed to determine the mass of sebum load, then separated and washed with 100 mL of surfactant solution. The samples were then thoroughly dried and rinsed with 20 mL of hexane together with the unwashed control sample. The hexane was evaporated after rinsing and the amount of sebum without washing and after washing with surfactant was weighed. The authors presented the detergent ability as a percentage of sebum removed and related it to synthetic detergents, which was 90.4%, 77.6%, and 60.0% of sebum removed for SDS, Tween 80, and saponin solution, respectively. The authors rated the cleaning properties of the saponins as moderate, where SDS performed much better. On the basis of the above-mentioned evaluation, surfactants from *S. mukorossi* have satisfactory washing ability and should be considered as a suitable detergent source.

### 6.7. Remediation Properties

The *Sapindus* species are used as phytoremediation plants, featured in afforestation and soil reclamation [24]. Mukhopadhyay et al. [98] evaluated the soil washing properties of saponins from *S. mukorossi* on zinc-contaminated soil at different parameters such as pH, surfactant concentration and soil: solution ratio, in relation to the synthetic surfactant SDS. Aqueous pericarp extracts at concentrations of 0.5, 1, 1.5, 2 and 2.5% (*w/v*) and aqueous SDS solutions of 10, 20 and 30 mM were used for soil washing. Soapnut showed greater zinc washing ability compared to SDS. The 2.5% solution of soapnut removed 73.54% zinc while the SDS 30 mM solutions only removed 31.45%. The reason was the difference in the pH of the surfactants, where the lower pH of the soapnut facilitated the removal of zinc. The study confirms the usefulness of *S. mukorossi* as a zinc washing agent from iron-rich soil with minimal damage to the soil. Mondal et al. [96] have assessed the uptake-reduction capabilities of hexavalent chromium contained in contaminated water, using saponins from the fruit pulp of *S. mukorossi*. Reduction in hexavalent chromium inside living cells is responsible for the formation of free radicals that cause permanent DNA damage. The authors extracted saponins using various solvents (pure water, pure ethanol, 50% aqueous ethanol, diethyl ether and methanol), obtaining the highest yield (77.4%) of saponins with a 50% (*v/v*) aqueous ethanol solution. The result of the experiment was presented in the form of a sorption capacity of 213.48 mg/g of hexavalent chromium, also determining the sorption parameters of the process, equal to pH 2 and temperature 35 °C. Kinetic analysis indicated that sorption is a spontaneous process.

## 7. Discussion and Remarks

*Sapindus mukorossi* is a rich source of many active compounds, including highly valued triterpenoid saponins. This plant is valued for its many traditional uses [30]. Widely used in folk medicine or as a source of natural surfactants, it has inherited the common name from its properties, washnut, *wu-huan-zi*, the ’non-illness fruit‘ [24]. Based on the cited studies, they clarify the reasons behind the great interest in the washnut plant in terms of biological and detergent-related properties. In addition, they explain in a scientific way the reasons for the practical use of raw materials from *S. mukorossi*. The main objective of the present review was to evaluate the properties of saponins from soapnut, in terms of the possible industrial application. 

Triterpenoid saponins contained in pericarp extracts exhibit antimicrobial activity against a wide range of both Gram-positive and Gram-negative pathogenic bacteria. Pericarp extracts showed strong inhibitory effects on bacterial growth in both in vitro and in vivo studies. In addition, continuous oral administration of the extract resulted in inhibition and treatment of bacterial infection in the tested rats [35,48,76,77]. Numerous studies also confirm the wide and potent antifungal activity of extracts and saponins isolated from *S. mukorossi* [22,76,77,78] as alternative sources of active compounds are constantly being sought as a substitute for synthetic drugs. The presented research results indicate that natural plant metabolites can be used in the fight against antibiotic-acquired microbial resistance [99]. Therefore, saponins may be a potential antimicrobial agent for use in the medical-pharmaceutical or food-cosmetic sectors [25,100]. 

Many other pharmaceutical properties described in the present paper, or described in more detail by other authors [27,30], explain the interest in the *S. mukorossi* tree in folk medicine. Various plant parts are used for therapeutic purposes [24]. Stem bark extracts and saponins isolated from the washnut show satisfactory in vitro and in vivo activity. When used as a drug on tested organisms, relative to commercial drugs, they can reverse the induced anti-inflammatory state [31,52,79]. Natural antioxidant properties are highly valued in the food and cosmetic industry [80]. Washnut extracts exhibited DPPH free radical, harmful oxide scavenging activity, and reducing properties. In this sense, they are suitable as protective agents for the natural oxidation of food and cosmetic products and for providing health protection against harmful radicals [31,80,81]. Further studies also indicate the antipyretic and analgesic activity of tree stem bark extracts on tested organisms [31]. The feature sought among active substances is cytotoxicity against cancer cells [101]. Leaf and stem extract and isolated saponins of the *S. mukorossi* tree showed activity against Hepa59T/VGH, NCI, HeLa, Med, and A549 cancer lines. In vivo tests on LL/2 cells of the extract produced no undesired organ damage, immunotoxicity, or off-target inflammation [34,41,46]. The active substances of the washnut plant have potential as an anticancer drug, thanks to which they can be used as a source of modern pharmaceuticals. On the basis of the above-mentioned evidence, the soapnut tree may be a promising pharmaceutical raw material. 

*S. mukorossi* is used by ethnic groups as a source of natural soap [26]. Saponins contained in the washnut can be used to prepare a natural herbal shampoo [102]. In biocosmetics, natural plant ingredients are widely used and carry many valuable biological properties [103]. Highlighting the previously mentioned antioxidant properties extracts from the seeds and fruit of soapnut show inhibition of tyrosinase, an enzyme crucial in tan formation. In addition, the saponin extracts also show inhibitory activity against the *P. acnes* bacteria responsible for skin and acne lesions. The extracts can therefore be used as a tan-removing agent to lighten the skin and treat acne lesions [61,80]. The seed oil also shows wound healing effects, the effectiveness of which has been evaluated in vitro and in vivo. More in-depth evaluation of healing activities is required to determine the medical application of the oil [37]. In this aspect, *S. mukorossi* seed oil is a valuable therapeutic resource, evaluated with skin-lightening, oral disease prevention, and dental regenerative properties [104,105,106]. Soapnut oil is also considered a suitable source of methyl esters, which can be used in biodiesel production [107].

It is a well-known fact that natural products, including plants, can exhibit toxicity. Therefore, it is important to assess the safety of products sourced from nature [108]. The inconclusive results of the toxicity assessment warrant the necessity of further studies. So far, studies have not shown dermal toxicity, but toxicity to aquatic organisms has been noted [61,84]. 

Soapnut saponins, either in isolated form or contained in extracts, show suitable detergent properties. The biodegradability of biosurfactants from the *S. mukorossi* tree is a very important feature of the plant [14]. Saponins present in extracts can be used as remediation agents for water and soil contaminated with chromium and zinc heavy metals [96,98]. Therefore, they may be used in environmental protection. They are also suitable as natural molluscicides and insecticides for the purpose of protecting plant crops from pests [59,74,82,83].

Detergents are an integral part of households and industrial areas. The properties of surfactants are classified between many functional characteristics. Among the most important are washing, foaming, emulsifying, solubilizing, or surface-active characteristics [8]. Soapnut saponins show adequate detergent properties to consider them as a potential substitute for synthetic surfactants. Reducing the surface tension of water from 72 mN/m to 35–32 mN/m is considered appropriate for suitable detergents [25]. The surface tension of aqueous washnut extracts varies between 52 and 35 mN/m [14,25,89,95,96]. The observed differences in tension may be dependent on the concentration, extraction methods, or raw material. With increasing concentrations of saponins, the surface tension decreases until it reaches a constant minimum for CMC, another parameter for evaluating the suitability of surfactants [14]. The lower the concentration of CMC, the easier micellization occurs, the micelles are more stable, and the surfactant shows better micellization ability [109]. The CMC of saponins obtained experimentally is within the range of 17–0.32 g/L [14,86,88,89,95] and 0.54 mmol/L [96]. It can be concluded that a lower mass input of saponins is necessary for micellization to occur in relation to the synthetic surfactants compared by the authors [14,86]. Again, CMC may depend on the source of saponins and extraction methods. 

The presence of micelles promotes the solubilization of substances that are heavily soluble in aqueous solutions [8]. When one considers emulsification-solubilization properties, in comparison to synthetic emulsifiers, one may observe that saponins were able to solubilize non-polar petroleum and vegetable derivatives in a similar manner and, in some cases, showed better properties than reference surfactants. Saponin emulsions were also characterized by satisfactory stability [14,86,95]. Detergents should also be expected to have stable, abundant foam and adequate wetting and washing properties [14]. The saponin-rich extracts showed excellent foaming properties. Compared to synthetic detergents, the foam was high and abundant, with long stability and resistance to higher temperatures [25,88]. The washing abilities of saponins were evaluated by simulating the washing process of previously stained fabrics with substances heavy to wash off. Natural surfactants were able to get rid of dirt from pieces of cloth to an appropriate degree, and the washing effect depended on the content of saponins. As they increased, the effects were noticeably better, matching those of synthetic detergents [14,97]. Saponins also moderately washed the fake sebum applied to human hair [25]. During the cleaning process, the wettability of the cleaning material, such as fabrics or surfaces, is essential. Suitable performance in this aspect allows for suitable wetting and penetration by liquid [110]. Washnut extracts wetted textile material and hydrophobic surfaces slightly worse than the synthetic surfactant. However, their utility should still be examined from this perspective, especially in favor of surface adsorption [25,88,89].

As natural metabolites, saponins are a biodegradable, non-toxic, and sustainable source of detergents [17]. On the basis of the examples cited, the practical use of the *Sapindus mukorossi* tree as an industrial raw material has been preliminarily summarized (Table 2.). However, the direct application of the substance in the mentioned fields requires a more in-depth investigation. The practical uses of the washnut tree are already being considered. In this case, *S. mukorossi* saponins have been evaluated as environmentally friendly coal dust suppressants [111], renewable latex polymerization additives [112], and foam-stabilizing agents in fire extinguishers [25].

## 8. Conclusions

*Sapindus mukorossi* is a source of valuable triterpenoid saponins, exhibiting a range of valuable properties. The plant also contains many other active compounds worthy of interest. Environmentally friendly saponins contained in the tree of the soapnut show a wide range of biological and detergent properties. Despite slightly worse experimental results, they should still be favored over synthetic surfactants, especially considering their natural origin, biodegradability, and complete renewability. In this respect, they perform well as raw materials, suitable for many branches of the cosmetic, food, pharmaceutical, and chemical industries. 

## Figures and Tables

**Table 1:** *Sapindus mukorossi* saponins present in different parts of the plant.

No.	Chemical Name	Abbreviations	Type	Ref.
**1**	3β,7β,20(*S*),22-tetrahydroxydammar-24-ene-3-*O*-α-l-rhamnopyranosyl-(1→2)-β-d-glucopyranoside	R_1_: -Glc_2_^a^-Rha ^b^ R_2_: -CH_3_ R_3_: -OH R_4_: -OH R_5_: -H	Dammarane	[41]
**2**	3β,7β,20(*S*),22,23-pentahydroxydammar-24-ene-3-*O*-α-l-rhamnopyranosyl-(1→2)-β-d-glucopyranoside	R_1_: -Glc_2_-Rha R_2_: -CH_3_ R_3_: -OH R_4_: -OH R_5_: -OH		
**3**	3β,7β,20(*S*),22,25-pentahydroxydammar-23-ene-3-*O*-α-l-rhamnopyranosyl-(1→2)-β-d-glucopyranoside	R_1_: -Glc_2_-Rha R_2_: -OH R_3_: -OH	Dammarane	[41]
**4**	25-methoxy-3β,7β,20(*S*),22-tetrahydroxydammar-23-ene-3-*O*-α-l-rhamnopyranosyl-(1→2)-β-d-glucopyranoside	R_1_: -Glc_2_-Rha R_2_: -OH R_3_: -OCH_3_		
**5**	25-methoxy-3β,7β,20(*R*)-trihydroxydammar-23-ene-3-*O*-α-l-rhamnopyranosyl-(1→2)-β-d-glucopyranoside	R_1_: -Glc_2_-Rha R_2_: -H R_3_: -OCH_3_		
**6**	21β-methoxy-3β,21(*S*),23(*R*)-epoxytirucalla-7,24-diene-3-*O*-α-l-rhamnopyranosyl-(1→6)-β-d-glucopyranoside	R_1_: -Glc_6_-Rha R_2_: β-OCH_3_ R_3_: β-H	Tirucallane	[40]
**7**	21α-methoxy-3β,21(*S*),23(*R*)-epoxytirucalla-7,24-diene-3-*O*-α-l-rhamnopyranosyl-(1→6)-β-d-glucopyranoside	R_1_: -Glc_6_-Rha R_2_: α-OCH_3_ R_3_: β-H		
**8**	21α-methoxy-3β,21(*R*),23(*R*)-epoxytirucalla-7,24-diene-3-*O*-α-l-rhamnopyranosyl-(1→2)-β-d-glucopyranoside	R_1_: -Glc_2_-Rha R_2_: α-OCH_3_ R_3_: β-H		
**9**	21β-methoxy-3β,21(*S*),23(*R*)-epoxytirucalla-7,24-diene-3-*O*-α-l-dirhamnopyranosyl-(1→2,6)-β-d-glucopyranoside	R_1_: -Glc_2,6_-Rha,Rha R_2_: β-OCH_3_ R_3_: β-H		
**10**	21α-methoxy-3β,21(*R*),23(*R*)-epoxytirucalla-7,24-diene-3-*O*-α-l-dirhamnonopyranosyl-(1→2,6)-β-d-glucopyranoside	R_1_: -Glc_2,6_-Rha,Rha R_2_: α-OCH_3_ R_3_: β-H		
**11**	Hederagenin-3-*O*-(3-*O*-acetyl-α-l-arabinopyranosyl)-(1→3)-α-l-rhamnopyranosyl-(1→2)-α-l-arabinopyranoside	R_1_: -Ara_2_-Rha_3_-Ara_3_^c^-OAc R_2_: -CH_2_OH R_3_: -H	Oleanane	[38]
**12**	Hederagenin-3-*O*-(4-*O*-acetyl-α-l-arabinopyranosyl)-(1→3)-α-l-rhamnopyranosyl-(1→2)-α-l-arabinopyranoside	R_1_: -Ara_2_-Rha_3_-Ara_4_-OAc R_2_: -CH_2_OH R_3_: -H		
**13**	Hederagenin-3-*O*-(2,3-*O*-diacetyl-β-d-xylopyranosyl)-(1→3)-α-l-rhamnopyranosyl-(1→2)-α-l-arabinopyranoside	R_1_: -Ara_2_-Rha_3_-Xyl_2,3_^d^-OAc,OAc R_2_: -CH_2_OH R_3_: -H		
**14**	Hederagenin-3-*O*-(2,4-*O*-diacetyl-β-d-xylopyranosyl)-(1→3)-α-l-rhamnopyranosyl-(1→2)-α-l-arabinopyranoside	R_1_: -Ara_2_-Rha_3_-Xyl_2,4_-OAc,OAc R_2_: -CH_2_OH R_3_: -H		
**15**	3,7,20(*S*)-trihydroxydammar-24-ene-3-*O*-α-l-rhamnopyrnosyl-(1→2)-β-d-glucopyranoside	R_1_: -Glc_2_-Rha R_2_: -OH R_3_: -CH_3_ R_4_: -H R_5_: -H	Dammarane	[38]
**16**	3,7,20(*R*)-trihydroxydammar-24-ene-3-*O*-α-l-rhamnopyrnosyl-(1→2)-β-d-glucopyranoside	R_1_: -Glc_2_-Rha R_2_: -CH_3_ R_3_: -OH R_4_: -H R_5_: -H		
**17**	21α-methoxy- 3β,21(*R*),23(*S*)-epoxytirucall-7,24-diene-3-*O*-β-d-glucopyranosyl-(1→2)-β-d-glucopyranoside	R_1_: -Glc_2_-Glc R_2_: α-OCH_3_ R_3_: β-H	Tirucallane	[39]
**18**	21α-methoxy-3β,21(*R*),23(*S*)-epoxytirucall-7,24-diene-3-*O*-α-l-rhamnopyranosyl-(1→6)-β-d-glucopyranosyl-(1→2)-β-d-glucopyranoside	R_1_: -Glc_2_-Glc_6_-Rha R_2_: α-OCH_3_ R_3_: β-H		
**19**	Hederagenin-3-*O*-(3,4-*O*-di-acetyl-β-d-xylopyranosyl)-(1→3)-α-l-rhamnopyranosyl-(1→2)-α-l-arabinopyranoside	R_1_: -Ara_2_-Rha_3_-Xyl_3_,_4_-OAc,OAc R_2_: -CH_2_OH R_3_: -H	Oleanane	[38]
**20**	Hederagenin-3-*O*-(2-*O*-acetyl-β-d-xylopyranosyl)-(1→3)-α-l-rhamnopyranosyl-(1→2)-α-l-arabinopyranoside	R_1_: -Ara_2_-Rha_3_-Xyl_2_-OAc R_2_: -CH_2_OH R_3_: -H		
**21**	Hederagenin-3-*O*-(3-*O*-acetyl-β-d-xylopyranosyl)-(1→3)-α-l-rhamnopyranosyl-(1→2)-α-l-arabinopyranoside	R_1_: -Ara_2_-Rha_3_-Xyl_3_-OAc R_2_: -CH_2_OH R_3_: -H		
**22**	Hederagenin-3-*O*-(4-*O*-acetyl-β-d-xylopyranosyl)-(1→3)-α-l-rhamnopyranosyl-(1→2)-α-l-arabinopyranoside	R_1_: -Ara_2_-Rha_3_-Xyl_4_-OAc R_2_: -CH_2_OH R_3_: -H		
**23**	Hederagenin-3-*O*-α-l-arabinopyranosyl-(1→3)-α-l-rhamnopyranosyl-(1→2)-α-l-arabinopyranoside	R_1_: -Ara_2_-Rha_3_-Ara R_2_: -CH_2_OH R_3_: -H		
**24**	21β-methoxy-3β,23α-epoxytirucalla-7,24-diene-3-*O*-α-l-rhamnopyranosyl-(1→6)-β-d-glucopyranoside	R_1_: -Glc_6_-Rha R_2_: β-OCH_3_ R_3_: α-H	Tirucallane	[42]
**25**	21β-methoxy-3β,23α-epoxytirucalla-7,24-diene-3-*O*-α-l-dirhamnopyranosyl-(1→2,6)-β-d-glucopyranoside	R_1_: -Glc_2,6_-Rha,Rha R_2_: β-OCH_3_ R_3_: α-H		
**26**	21α-methoxy-3β,23α-epoxytirucalla-7,24-diene-3-*O*-α-l-rhamnopyranosyl-(1→2)-[α-l-arabinopyranosyl-(1→3)]-β-d-glucopyranoside	R_1_: -Glc_2,3_-Rha,Ara R_2_: α-OCH_3_ R_3_: α-H		
**27**	21α-methoxy-3β,23α-epoxytirucalla-7,24-diene-3-*O*-α-l-rhamnopyranosyl-(1→2)-β-d-glucopyranoside	R_1_: -Glc_2_-Rha R_2_: α-OCH_3_ R_3_: α-H		
**28**	3-*O*-α-l-rhamnopyranosyl-(1→2)-[α-l-arabinopyranosyl-(1→3)]-β-d-glucopyranosyl-21,23*R*-epoxyl tirucall-7,24*R*-diene-3β,21-diol	R_1_: -Glc_2,3_-Rha,Ara R_2_: -OH	Tirucallane	[44]
**29**	3-*O*-α-l-rhamnopyranosyl-(1→6)-β-d-glucopyranosyl-21,23R-epoxyl tirucall-7,24R-diene-3β,21-diol	R_1_: -Glc_6_-Rha R_2_: -OH		
**30**	3-*O*-α-l-rhamnopyranosyl-(1→2)-[α-l-arabinopyranosyl-(1→3)]-β-d-glucopyranosyl (21,23*R*)-epoxyl tirucalla-7,24-diene-(21*S*)-ethoxyl-3β-ol	R_1_: -Glc_2,3_-Rha,Ara R_2_: -OCH_2_CH_3_	Tirucallane	[45]
**31**	3-*O*-α-l-rhamnopyranosyl-(1→2)-[α-l-arabinopyranosyl-(1→3)]-β-d-glucopyranosyl (21,23*R*)-epoxyl tirucall-7,24-diene-(21*S*)-methoxyl-3β-ol	R_1_: -Glc_2,3_-Rha,Ara R_2_: -OCH_3_		
**32**	3-*O*-α-l-arabinopyranosyl-(1→3)-α-l-rhamnopyranosyl-(1→2)-[α-l-arabinopyranosyl-(1→3)]-β-d-glucopyranosyl-21,23R-epoxyl tirucalla-7,24-diene-21β-ethoxy-3β-ol	R_1_: -Glc_2,3_-(Rha_3_-Ara),Ara R_2_: -OCH_2_CH_3_	Tirucallane	[43]
**33**	3-*O*-β-d-xylopyranosyl-(1→3)-α-l-rhamnopyranosyl-(1→2)-[β-l-arabinopyranosyl-(1→3)]-β-d-glucopyranosyl-21,23*R*-epoxyl tirucalla-7,24-diene-21β-ethoxy-3β-ol	R_1_: -Glc_2,3_-(Rha_3_-Xyl),Ara R_2_: -OCH_2_CH_3_		
**34**	3-*O*-β-d-xylopyranosyl-(1→3)-α-l-rhamnopyranosyl-(1→2)-[α-l-arabinopyranosyl-(1→3)]-β-d-glucopyranosyl-21,23R-epoxyl tirucalla-7,24-diene-21β-methoxy-3β-ol	R_1_: -Glc_2,3_-(Rha_3_-Xyl),Ara R_2_: -OCH_3_		
**35**	3-*O*-α-l-arabinopyranosyl-(1→3)-α-l-rhamnopyranosyl-(1→2)-[α-l- rhamnopyranosyl-(1→3)]-β-d-glucopyranosyl-21,23*R*-epoxyl tirucalla-7,24-diene-21β-ethoxy-3β-ol	R_1_: -Glc_2,3_-(Rha_3_-Ara),Rha R_2_: -OCH_2_CH_3_		
**36**	3-*O*-α-l-arabinopyranosyl-(1→3)-α-l-rhamnopyranosyl-(1→2)-[α-l-rhamnopyranosyl-(1→3)]-β-d-glucopyranosyl-21,23R-epoxyl tirucalla-7,24-diene-21β-methoxy-3β-ol	R_1_: -Glc_2,3_-(Rha_3_-Ara),Rha R_2_: -OCH_3_		
**37**	3-*O*-α-l-rhamnopyranosyl-(1→6)-β-d-glucopyranosyl-21,23R-epoxyl tirucalla-7,24-diene-21β-ethoxyl-3β-ol	R_1_: -Glc_6_-Rha R_2_: -OCH_2_CH_3_		
**38**	Hederagenin-3-*O*-β-d-xylopyranosyl-(1→3)-α-l-rhamnopyranosyl-(1→2)-α-l-arabinopyranosyl-28-*O*-β-d-glucopyranosyl-(1→2)-β-d-glucopyranosyl ester	R_1_: -Ara_2_-Rha_3_-Xyl R_2_: -CH_2_OH R_3_: -Glc_2_-Glc	Oleanane	[47]
**39**	Hederagenin-3-*O*-α-l-arabinopyranosyl-(1→3)-α-l-rhamnopyranosyl-(1→2)-α-l-arabinopyranosyl-28-*O*-β-d-glucopyranosyl-(1→2)-β-d-glucopyranosyl ester	R_1_: -Ara_2_-Rha_3_-Ara R_2_: -CH_2_OH R_3_: -Glc_2_-Glc		
**40**	Hederagenin-3-*O*-α-l-rhamnopyranosyl-(1→2)-α-l-arabinopyranosyl-28-*O*-β-d-glucopyranosyl-(1→2)-β-d-glucopyranosyl ester	R_1_: -Ara_2_-Rha R_2_: -CH_2_OH R_3_: -Glc_2_-Glc		
**41**	Hederagenin-3-*O*-α-l-rhamnopyranosyl-(1→2)-α-l-arabinopyranoside	R_1_: -Ara_2_-Rha R_2_: -CH_2_OH R_3_: -H	Oleanane	[54]
**42**	Hederagenin-3-*O*-β-d-xylopyranosyl-(1→3)-α-l-rhamnopyranosyl-(1→2)-α-l-arabinopyranoside	R_1_: -Ara_2_-Rha_3_-Xyl R_2_: -CH_2_OH R_3_: -H		
**43**	Hederagenin-3-*O*-β-d-glucopyranosyl-(1→4)-β-d-xylopyranosyl-(1→3)-α-l-rhamnopyranosyl-(1→2)-α-l-arabinopyranoside	R_1_: -Ara_2_-Rha_3_-Xyl_4_-Glc R_2_: -CH_2_OH R_3_: -H	Oleanane	[57]
**44**	Hederagenin-3-*O*-β-d-glucopyranosyl-(1→2)-[α-l-rhamnopyranosyl-(1→6)]-β-d-glucopyranosyl-(1→4)-β-d-xylopyranosyl-(1→3)-α-l-rhamnopyranosyl-(1→2)-α-l-arabinopyranoside	R_1_: -Ara_2_-Rha_3_-Xyl_4_-Glc_2,6_-Glc,Rha R_2_: -CH_2_OH R_3_: -H	Oleanane	[56]
**45**	Hederagenin-3-*O*-β-d-xylopyranosyl-(1→3)-α-l-rhamnopyranosyl-(1→2)-α-l-arabinopyranosyl-28-*O*-β-d-glucopyranosyl-(1→2)-[α-l-rhamnopyranosyl-(1→6)]-β-d-glucopyranosyl-(1→4)-β-d-xylopyranosyl-(1→3)-α-l-rhamnopyranosy-(1→2)-α-l-arabinopyranosyl ester	R_1_: -Ara_2_-Rha_3_-Xyl R_2_: -CH_2_OH R_3_: -Ara_2_-Rha_3_-Xyl_4_-Glc_2,6_-Glc,Rha	Oleanane	[55]
**46**	Hederagenin-3-*O*-β-d-glucopyranosyl-(1→3)-β-d-xylopyranosyl-(1→3)- β-d-xylopyranosyl-(1→3)-α-l-rhamnopyranosyl-(1→2)-α-l-arabinopyranoside	R_1_: -Ara_2_-Rha_3_-Xyl_3_-Xyl_3_-Glc R_2_: -CH_2_OH R_3_: -H	Oleanane	[58]
**47**	Hederagenin-3-*O*-(3,4-*O*-diacetyl-α-l-arabinopyranosyl)-(1→3)-α-l-rhamnopyranosyl-(1→2)-α-l-arabinopyranoside	R_1_: -Ara_2_-Rha_3_-Ara_3,4_-OAc,OAc R_2_: -CH_2_OH R_3_: -H		
**48**	3-*O*-α-l-rhamnopyranosyl-(1→2)-β-d-xylopyranosyl-(1→6)-β-d-glucopyranosyl-(1→3)-β-d-xylopyranosyl-(1→3)-α-l-rhamnopyranosyl-(1→2)-α-l-arabinopyranosyl oleanolic acid	R_1_: -Ara_2_-Rha_3_-Xyl_3_-Glc_6_-Xyl_2_-Rha R_2_: -CH_3_ R_3_: -H	Oleanane	[46]
**49**	Hederagenin 3-*O*-(2,4-*O*-di-acetyl-α-l-arabinopyranosyl)-(1→3)-α-l-rhamnopyranosyl-(1→2)-α-l-arabinopyranoside	R_1_: -Ara_2_-Rha_3_-Ara_2,4_-OAc,OAc R_2_: -CH_2_OH R_3_: -H	Oleanane	[59]
**50**	Hederagenin 3-*O*-α-l-arabinopyranoside	R_1_: -Ara R_2_: -CH_2_OH R_3_: -H		
**51**	Hederagenin-3-*O*-β-d-xylopyranosyl-(2→1)-[3-*O*-acetyl-α-l-arabinopyranosyl]-28-*O*-α-l-rhamnopyranosylester	R_1_: -Xyl_2_-Ara_3_-OAc R_2_: -CH_2_OH R_3_: -Rha	Oleanane	[48]
**52**	Hederagenin 3-*O*-α-l-rhamnopyranosyl (3→1)-[2,4-*O*-diacetyl-α-l-arabinopyranosyl]-28-*O*-β-d-glucopyranosyl-(2→1) [3-*O*-acetyl-β-d-glucopyranosyl] ester	R_1_: -Rha_3_-Ara_2,4_-OAc,OAc R_2_: -CH_2_OH R_3_: -Glc_2_-Glc_3_-OAc	Oleanane	[50]
**53**	Oleanolic acid 3-*O*-α-l-arabinofuranosyl-(1→3)-α-l-rhamnopyranosyl-(1→2)-α-l-arabinopyranoside	R_1_: -Ara_2_-Rha_3_-Ara ^e^ R_2_: -CH_3_ R_3_: -H	Oleanane	[22]
**54**	Hederagenin 3-*O*-5‴-*O*-acetyl-α-l-arabinofuranosyl-(1→3)-α-l-rhamnopyranosyl-(1→2)-α-l-arabinopyranoside	R_1_: -Ara_2_-Rha_3_-Ara_5_^e^-OAc R_2_: -CH_2_OH R_3_: -H		
**55**	23-*O*-acetyl-hederagenin 3-*O*-β-d-xylopyranosyl-(1→3)-α-l-rhamnopyranosyl-(1→2)-α-l-arabinopyranoside	R_1_: -Ara_2_-Rha_3_-Xyl R_2_: -CH_2_OAc R_3_: -H		
**56**	Gypsogenin 3-*O*-α-l-arabinopyranosyl-(1→3)-α-l-rhamnopyranosyl-(1→2)-α-l-arabinopyranoside	R_1_: -Ara_2_-Rha_3_-Ara R_2_: -CH_2_O R_3_: -H		
**57**	Betulinic acid 3-*O*-β-d-xylopyranosyl-(1→3)-α-l-rhamnopyranosyl-(1→2)-α-l-arabinopyranoside	R_1_: -Ara_2_-Rha_3_-Xyl	Lupane	[22]
**58**	Hederagenin-3-*O*-β-d-glucopyranosyl-(1→2)-α-l-arabinopyranoside	R_1_: -Ara_2_-Glc R_2_: -CH_2_OH R_2_: -H	Oleanane	[36]
**59**	Hederagenin-3-*O*-α-l-rhamnopyranosyl-(1→3)-α-l-rhamnopyranosyl-(1→2)-α-l-arabinopyranoside	R_1_: -Ara_2_-Rha_3_-Rha R_2_: -CH_2_OH R_3_: -H	Oleanane	[36]
**60**	Hederagenin-3-*O*-β-d-xylopyranosyl-(1→3)-α-l-arabinopyranoside	R_1_: -Ara_2_-Xyl R_2_: -CH_2_OH R_3_: -H		
**61**	Hederagenin-3-*O*-(4-*O*-acetyl-β-d-glucopyranosyl)-(1→3)-α-l-rhamnopyranosyl-(1→2)-α-l-arabinopyranoside	R_1_: -Ara_2_-Rha_3_-Glc_4_-OAc R_2_: -CH_2_OH R_3_: -H		
**62**	3-*O*-β-d-glucopyranosyl-(1→2)-α-l-rhamnopyranosyl-(1→3)-α-l-rhamnopyranosyl-(1→2)-β-d-glucopyranosyl oleanolic acid	R_1_: -Glc_2_-Rha_3_-Rha_2_-Glc R_2_: -CH_3_ R_3_: -H		
**63**	3-*O*-β-d-xylopyranosyl-(1→2)-α-l-rhamnopyranosyl-(1→3)-α-l-rhamnopyranosyl-(1→2)-β-d-glucopyranosyl oleanolic acid	R_1_: -Glc_2_-Rha_3_-Rha_2_-Xyl R_2_: -CH_3_ R_3_: -H		
**64**	Oleanolic acid 3-*O*-(4-*O*-acetyl-α-l-arabinopyranosyl)-(1→3)-α-l-rhamnopyranosyl-(1→2)-α-l-arabinopyranoside	R_1_: -Ara_2_-Rha_3_-Ara_4_-OAc R_2_: -CH_3_ R_3_: -H		
**65**	Gypsogenin 3-O-α-l-rhamnopyranosyl-(1→3)-α-l-rhamnopyranosyl-(1→2)-α-l-arabinopyranoside	R_1_: -Ara_2_-Rha_3_-Rha R_2_: -CHO R_3_: -H		
**66**	Oleanolic acid 3-*O*-β-d-xylopyranosyl-(1→3)-α-l-rhamnopyranosyl-(1→2)-α-l-arabinopyranoside	R_1_: -Ara_2_-Rha_3_-Xyl R_2_: -CH_3_ R_3_: -H	Oleanane	[22]
**67**	Oleanolic acid 3-*O*-α-l-arabinopyranosyl-(1→3)-α-l-rhamnopyranosyl-(1→2)-α-l-arabinopyranoside	R_1_: -Ara_2_-Rha_3_-Ara R_2_: -CH_3_ R_3_: -H		

^a^ β-d-Glucopyranosyl, ^b^ α-l-Rhamnopyranosyl, ^c^ α-l-Arabinopyranosyl, ^d^ β-d-Xylopyranosyl, ^e^ α-l-Arabinofuranosyl.

**Table 2:** Preliminary assessment of the purpose of *Sapindus mukorossi* as an industrial raw material.

Sapindus mukorossi Soapnut Tree	Properties	Purpose
Extracts, Oils and Isolated Saponins	Antibacterial and Antifungal	Preservatives, Disinfectants, Antibiotics
	Tyrosinase and *P. acnes* inhibitor	Skin whitening, Anti-acne cosmetics
	Antioxidant, Wound healing	Natural food and cosmetic antioxidants, Skin-care cosmetics
	Antitumor and Cytotoxic	Natural anticancer drugs
	Molluscicidal and Insecticidal	Natural pesticides, Plant protection products
	Antipyretic, Analgesic, Anti-inflammatory	Herbal painkillers, Anti-inflammatory, and Antipyretic drugs
	Surface tension reduction, Foaming, Wetting, Washing	Natural detergents, Wetting, Washing, Foaming, and Cleaning agents
	Micelle formation, Solubilization, and Leaching	Natural solubilizers, Emulgators, Leaching, and Remediation agents

## Data Availability

Not applicable.
